# Incidence of Mutations in the *ALPL*, *GGPS1*, and *CYP1A1* Genes in Patients With Atypical Femoral Fractures

**DOI:** 10.1002/jbm4.10064

**Published:** 2018-06-22

**Authors:** Pilar Peris, Eva González‐Roca, Sebastian C Rodríguez‐García, María del Mar López‐Cobo, Ana Monegal, Núria Guañabens

**Affiliations:** ^1^ Rheumatology Department Institut d'Investigacions Biomèdiques August Pi i Sunyer (IDIBAPS) Network for Biomedical Research in Hepatic and Digestive Diseases (CIBERehd) Clínic Foundation for Biomedical Research (FCRB) Hospital Clínic University of Barcelona Barcelona Spain; ^2^ Immunology Department IDIBAPS CIBERehd FCRB Hospital Clínic University of Barcelona Barcelona Spain; ^3^ Molecular Biology Core Laboratory IDIBAPS CIBERehd FCRB Hospital Clínic University of Barcelona Barcelona Spain

**Keywords:** ATYPICAL FEMORAL FRACTURES, HYPOPHOSPHATASIA, GENE MUTATIONS, BISPHOSPHONATES, *ALPL*, *GGPS1*, *CYP1A1*

## Abstract

Atypical femoral fractures (AFFs) are uncommon and often related to prolonged bisphosphonate (BP) treatment. Isolated cases have been linked to mutations of tissue nonspecific alkaline phosphatase (*ALPL*). Moreover, mutations in the geranylgeranyl pyrophosphate synthase (*GGPPS*) gene, which can be inhibited by BPs, and in the enzyme of the cytochrome P450 superfamily (*CYP1A1*), related to the metabolism of several drugs, have also been associated with AFF development. Our aim was to analyze the incidence of *ALPL*, *GGPS1*, and *CYP1A1* gene mutations in patients with AFFs and their clinical characteristics. Seventeen women with AAFs were included. All patients underwent Sanger sequencing of the *ALPL*, *GGPS1*, and *CYP1A1* genes, analyzing the presence of mutations and polymorphisms in these genes. The clinical characteristics of the patients, previous treatments, ALP substrates (vitamin B6 and phosphoethanolamine), bone turnover markers, and bone mass were also analyzed. Three of 17 patients (17.6%) presented heterozygous mutations in the *ALPL* (p.Gly288Ala) or *CYP1A1* (p.Arg136His, p.Val409Ile) genes. Only the patient with the *ALPL* mutation presented increased ALP substrates. Patients with *CYP1A1* variants had glucocorticoid‐induced osteoporosis. All patients were previously treated with BPs during 85.5 ± 38 months, and nearly 50% were also treated with glucocorticoids. The AFF was bilateral in 35% of cases. In conclusion, *ALPL* and *CYP1A1* mutations may be related to the development of AFF in patients treated with BPs. The evaluation of ALP substrates in patients with low ALPL levels allows the identification of patients with hypophosphatasia. The role of *CYP1A1* mutations in AFF needs further study. © 2018 The Authors. *JBMR Plus* published by Wiley Periodicals, Inc. on behalf of American Society for Bone and Mineral Research.

## Introduction

Atypical femoral fractures (AFFs) are an uncommon type of fracture often related to prolonged bisphosphonate (BP) treatment. This type of fracture is commonly nontraumatic. It is characteristically located in the subtrochanteric region or the femoral diaphysis and is frequently bilateral. Although the cause of AFF is not well known, its development has been linked to long‐term use of BPs.^1^, ^2^, ^3^, ^4^ However, isolated cases of AFF have also been related to mutations of tissue nonspecific alkaline phosphatase (*ALPL*) as a clinical form of presentation of hypophosphatasia in adults.^5^, ^6^

The clinical presentation of hypophosphatasia may vary widely, from severe perinatal to mild forms with scarce clinical expression in adults.^7^ This entity may be easily overlooked in adults presenting with stress fractures in the lower extremities, articular chondrocalcinosis, or relapsing calcific periarthritis.^8^, ^9^ Clinical suspicion is therefore essential for diagnosis. In addition, a recent study described mutations in the enzyme of geranylgeranyl pyrophosphate synthase (*GGPS1*), which can be inhibited by BPs, as well as in the enzyme of the cytochrome P450 superfamily *CYP1A1*, the latter related to the metabolism of several drugs associated with the development of AFF.^10^, ^11^ Taking into account all of the above and the recent recommendations of the AFF Task Force of the American Society for Bone and Mineral Research (ASBMR) regarding the need for greater knowledge on the pathophysiology of AFF,^3^ it is essential to know if subjects with AFF present genetic alterations favoring the development of this characteristic type of fracture.

Therefore, the aim of this study was to analyze the presence of *ALPL*, *GGPS1*, and *CYP1A1* gene mutations in patients with AFF as well as the clinical characteristics of these patients.

## Patients and Methods

### Patients

We included all the patients diagnosed with AFF in our hospital from January 2009 to January 2016. The study was conducted at the Metabolic Bone Diseases Unit of the Rheumatology Department of the Hospital Clinic. The diagnosis of AFF was based on the ASBMR task force criteria.^3^ In all subjects a clinical history was obtained with special reference to risk factors for osteoporosis and previous symptoms of AFF. Previous skeletal fractures, age at menopause, and previous treatments for osteoporosis and duration were recorded in all patients, as well as weight, height, and body mass index (BMI) expressed as weight per height squared (kg/m^2^). In addition, other concomitant treatments and diseases were also recorded. All subjects gave informed consent, and the Ethics Committee of the hospital approved the study (HCB/2014/0045). After acceptance and signing the informed consent to participate in the study, blood analysis for genetic and biochemical studies and BMD measurements were performed. Patients were clinically assessed after presenting the fracture.

### Biochemical determinations

The biochemical profile at baseline included: serum creatinine, glucose, calcium, and phosphate, total alkaline phosphatase (total ALP) performed by standard procedures; the substrates of alkaline phosphatase (pyridoxal‐5′ phosphate in serum [vitamin B6] and phosphoethanolamine in urine [PEA]) (performed by high‐pressure liquid chromatography); serum 25OHD (using the Liason DiaSorin, chemiluminiscent immunoassay system, Stillwater, MN, USA); bone alkaline phosphatase (bone ALP) and propeptide amino‐terminal of type I procollagen (P1NP) as bone formation markers assessed by ELISA (IDS, Vitro, Boldon, England) and electrochemiluminescence by the automated Cobas e411 method (Roche, Mannheim, Germany); and serum carboxy‐terminal telopeptide of type I collagen (sCTx) by the automated Cobas e411 method (Roche), the latter determined as a bone resorption marker. Blood and urinary samples were obtained between 8:00 a.m. and 10:00 a.m. after overnight fasting.

### Genetic analysis

Sanger sequencing for the *ALPL*, *GGPS1*, and *CYP1A1* genes was performed in all patients. Genomic DNA was extracted from peripheral blood using the MagNA Pure 96 DNA and Viral NA Large Volume Kit on the automated DNA extractor MagNA Pure 96 System (Roche Life Science, Switzerland). Standard PCR procedures were performed for Sanger sequencing of all the exons and intron splicing sites of the *ALPL* (NM_000478), *GGPS1* (NM_001037277), and *CYP1A1* (NM_000499) genes. Primer sequences are available upon request.

### BMD measurements

BMD of the proximal femur (neck and total femur) and lumbar spine was measured by dual X‐ray absorptiometry (Lunar Prodigy, Radiation Corporation Madison, WI, USA). The coefficients of variations for total femur and lumbar spine are 0.6 and 0.8, respectively. Osteoporosis was defined according to the WHO criteria with *T*‐score values < −2.5 in any of these locations.^12^

### Data analysis

All data are expressed as mean ± standard deviation (SD). The nonparametric Mann‐Whitney *U* test was used to compare differences for continuous variables. Differences between proportions were assessed with the chi‐square test. A *p* value <0.05 was considered statistically significant.

Genetic data analysis was performed with the SeqPilot module of the JSI medical System GmbH software. Sorting Tolerant from Intolerant (SIFT), Polyphen2, and Mutation Taster algorithms were used to predict the effect of the new or low‐frequency variants detected in patients’ samples.^13^, ^14^, ^15^ Classification of variants into pathogenic, uncertain significance, or benign was performed following the American College of Medical Genetics and Genomics (ACMG) recommendations.^16^ Visualization of the *ALPL* variant in the 3D model of Alpl protein was performed using PyMol v0.98rc5 visualization software with the model described by Silvent and colleagues.^17^

## Results

### General characteristics

Seventeen patients (all white women) with AAF with a mean age of 71 ± 10 years (range, 52 to 87 years) were included in the study. The characteristics of the patients are shown in Table 1. Briefly, all patients (17/17) were previously treated with BPs during a mean period of 85.5 ± 38 months (range, 14 to 144 months); most (16/17) received treatment with alendronate (3/16 cases received sequential treatment: alendronate followed by zoledronate [one case] or denosumab [two cases]), and only one of 17 was treated with risedronate. Treatment with BPs was ≤5 years in five patients. Most patients received calcium and vitamin D supplementation (15/17; 88%). Nearly 50% of the patients (8/17) were additionally treated with glucocorticoids (GCC), most cases for rheumatologic disorders (Table 1); 18% of the AFF patients (3/17) were diabetic and 47% (7/17) received concomitant treatment with proton pump inhibitors (PPIs). Most patients (13/17; 76%) had had previous fragility fractures and 55% (6/11 with BMD available for hip and/or spine) had densitometric osteoporosis. In relation to the characteristics of AFF, most patients presented the fracture in the right femur (10/17), only one in the left, being the AFF bilateral in six cases (35%). Most patients (13/17; 76%) had a spontaneous fracture with only four patients presenting the fracture after a low‐energy trauma. Previous symptoms, in the form of groin or thigh pain, were reported in 41% of the patients (7/17), with a mean duration of 6 ± 2.8 months.

**Table 1:** General Characteristics of the Patients With AFFs

Case	Sex (M/F)/ Age (years)	Underlying disease	GCC therapy	BMI (kg/m^2^)	Previous OP therapy^a^	Duration of anti‐OP treatment (months)	Previous fragility Fx	Treatment with PPIs	Diabetes	Type of AFF	Presence of previous associated symptoms related to the fracture	Duration of symptoms (months)	FN *T*‐score
1	F/63	GCC‐OP; ITP	Yes	29.9	ALD	96	Yes	No	No	Bilateral	No	–	−2
2	F/71	PM‐OP	No	29.2	ALD	72	Yes	Yes	No	Bilateral	No	–	−2.3
					Dmab	12							
3	F/75	GCC‐OP; PMR	Yes	33.7	RIS	60	Yes	Yes	No	Unilateral	No	–	−2.5
4^b^	F/77	GCC‐OP; COPD	Yes	32.3	ALD	36	Yes	Yes	Yes	Unilateral	Yes	2	−2.7
5	F/77	PM‐OP	No	22.8	ALD	72	Yes	No	No	Unilateral	Yes	2	−1.8
6	F/68	GCC‐OP; asthma	Yes	24.6	ALD	132	Yes	–	No	Unilateral	No	–	−2
7^b^	F/67	PM‐OPc	No	23.7	ALD	96	No	No	No	Unilateral	Yes	3	−2.2
8	F/52	GCC‐OP; RA	Yes	29.1	ALD	132	No	No	No	Bilateral	Yes	12	−2.3
9	F/78	PM‐OP	No	29.9	ALD	72	Yes	No	No	Bilateral	Yes	24	–
10	F/56	GCC‐OP; Sjögren syndrome; sarcoidosis	Yes	26.1	ALD	120	Yes	Yes	No	Unilateral	No	–	−1.5
					ZOL	24							
11	F/76	PM‐OP	No	35.1	ALD	36	Yes	No	No	Unilateral	Yes	3	−1
					Dmab	6							
12	F/52	GCC‐OP; polymyositis	Yes	26.3	ALD	120	Yes	Yes	No	Unilateral	No	–	−2.3
13	F/83	PM‐OP	No	36.7	ALD	48	Yes	No	Yes	Unilateral	No	–	−2.6
14	F/69	PM‐OP	No	26.2	ALD	14	Yes	Yes	Yes	Unilateral	No	–	–
15	F/81	PM‐OP	No	28.5	ALD	96	No	No	No	Bilateral	No	–	–
16	F/87	PM‐OP	No	28	ALD	108	No	No	No	Bilateral	Yes	2	–
17^b^	F/75	GCC‐OP; RA	Yes	29.1	ALD	144	Yes	Yes	No	Unilateral	No	–	−1.3

AFF = atypical femoral fracture; M = male; F = female; GCC = glucocorticoid; BMI = body mass index; OP = osteoporosis; Fx = fracture; PPI = proton pump inhibitor; FN = femoral neck; GCC‐OP = glucocorticoid‐induced osteoporosis; ITP = idiopathic thrombocytopenic purpura; ALD = alendronate; PM‐OP = postmenopausal osteoporosis; Dmab = denosumab; PMR = polymyalgia rheumatica; RIS = risedronate; COPD = chronic obstructive pulmonary disease; RA = rheumatoid arthritis; ZOL = zoledronate.

^a^ Previous treatment of OP with ALD, RIS, ZOL, or Dmab.

^b^ The three patients with gene mutations (cases 4 and 17: CYP1A1 mutation; case 7: ALPL mutation).

^c^Initially diagnosed with PM‐OP but after the fracture the diagnosis was hypophosphatasia.

Vitamin D serum levels were deficient (<20 ng/mL) in most of the patients (8/11) analyzed after the fracture (73%), but none of the patients presented biochemical alterations consistent with osteomalacia (data not shown).^18^ Formation and/or resorption bone turnover markers were among the reference values in most patients, with increased values in 44% (7/16) and 21% (3/14), respectively (Table 2).

**Table 2:** Variants Identified in the *ALPL*, *GGPS1*, and *CYP1A1* Genes and Laboratory Results in Patients With Atypical Femoral Fractures

	Variants													
Case	Gene	Nucleotide change	Amino acid change	Status	Variant classification^a^	Variant id	ExAC frequencies (EUR–Non‐Finnish) (%)^b^	Bioinformatic predictions^c^	Total ALP^d^	Vitamin B6^e^	Bone ALP^f^	P1NP^g^	CTx^h^	25OHD^i^
1	*ALPL*	c.455G>A	p.Arg152His	Heterozygous	Pol	rs14934498	1.16		97	36	7.1	10	0.14	28.5
2	*GGPS1*	c.142‐6dup	−	Heterozygous	Pol	Rs3841735	38.1		−	10	−	−	−	−
3	−	−	−	−	−				132*	18	−	36	−	18.6
4^j^	*CYP1A1*	c.407G>A	p.Arg136His	Heterozygous	VUS	rs202201538	0*	Damaging; benign; disease causing	97	45	13.5	39	0.11	15.4
	*GGPS1*	c.142‐6dup	−	Heterozygous	Pol	rs3841735	38.1							
5	−	−	−	−	−				74	40	**14.9**	**66**	0.41	16.7
6	*ALPL*	c.1565T>C	p.Val522Ala	Homozygous	Pol	rs34605986	12.39		78	36	9.7	20	0.11	22.4
	*GGPS1*	c.142‐6dup	–	Homozygous	Pol	rs3841735	38.1							
7^j^	*ALPL*	c.863G>C	p.Gly288Ala	Heterozygous	Likely pathogenic		NA	Damaging; probably damaging; disease causing	97*	**143**	10.2	**67**	**0.71**	−
	*GGPS1*	c.142‐6dup	−	Heterozygous	Pol	rs3841735	38.1							
8	*ALPL*	c.1565T>C	p.Val522Ala	Heterozygous	Pol	rs34605986	12.39		84	−	10.5	8	0.16	−
9	*ALPL*	c.1565T>C	p.Val522Ala	Heterozygous	Pol	rs34605986	12.39		50	61	−	49	−	18
	*GGPS1*	c.142‐6dup	–	Homozygous	Pol	rs3841735	38.1							
10	*ALPL*	c.1565T>C	p.Val522Ala	Heterozygous	Pol	rs34605986	12.39		218*	24	24	53	0.20	−
11	*ALPL*	c.455G>A	p.Arg152His	Heterozygous	Pol	rs14934498	1.16		92	17	**17.3**	**86**	0.27	−
12	*GGPS1*	c.142‐6dup	−	Homozygous	Pol	rs3841735	38.1		120	17	10.6	35	0.29	16.2
13	*ALPL*	c.787T>C	p.Tyr263His	Homozygous	Pol	rs3200254	10.76		94	26	**25.8**	**71**	0.52	100
	*GGPS1*	c.142‐6dup	−	Heterozygous	Pol	rs3841735	38.1							
14	−	−	−	−	−	−			211	38	**33.7**	**94**	**0.81**	10
15	*GGPS1*	c.142‐6dup	−	Homozygous	Pol	rs3841735	38.1		108	72	−	**162**	0.56	16.4
16	*ALPL*	c.455G>A	p.Arg152His	Heterozygous	Pol	rs14934498	1.16		82	21	−	**93**	**0.65**	17.8
	*CYP1A1*	c.1382C>A	p.Thr461Asn	Heterozygous	Pol	rs1799814	4.4							
17j	*CYP1A1*	c.1225G>A	p.Val409Ile	Heterozygous	VUS	rs769134905	NA	Tolerated; benign; disease‐causing	54	26	7.7	25	0.29	−

Values in bold are above the normal range.

ExAC = Exome Aggregation Consortium; EUR = European; ALP = alkaline phosphatase; Pol = polymorphism; VUS = variant of uncertain significance.

^a^ Classification of variants following the ACMG recommendations.^16^

^b^ ExAC frequencies: only European–Non‐Finnish population frequencies shown (NA: not registered in ExAC; *8.651e–05 in Latino population [1 allele out of 11560]).

^c^ Predictions from SIFT, Polyphen2, and MutationTaster, respectively.

^d^ Total ALP (normal values: 46–116 U/L; *measured in some patients with another method: normal values: 80–240 U/L).

^e^ Vitamin B6 (normal values: 15–96 nmol/L).

^f^ Bone ALP (reference values in premenopausal women: 6–13.6 ng/mL).

^g^ P1NP (reference values in premenopausal women: 22–63 ng/mL).

^h^ CTx (reference values in premenopausal women: 0.02–0.58 ng/mL).

^i^ 25 OH vitamin D (normal values: ≥20 ng/mL).

^j^ The three patients with gene mutations (cases 4 and 17: CYP1A1 mutation; case 7: ALPL mutation).

When comparing patients with AFF treated with versus without GCC, the former were younger (64 ± 75 versus 76 ± 56 years, *p* = 0.015) and were also treated with BPs for a longer period of time (105 ± 38 versus 68.2 ± 30 months, *p* = 0.046). These patients also showed significantly lower mean values of bone formation and resorption markers (Table 3).

**Table 3:** Patients With AFFs Associated With GCC Treatment Versus Non–GCC‐Treated Patients

	GCC AFF (*n* = 8)	No GCC AFF (*n* = 9)	*p*
Age (years)	64 ± 75	76 ± 56	0.015
BMI (kg/m^2^)	28.9 ± 3.1	28.9 ± 4.6	n.s
Duration of BP treatment (months)	105 ± 38	68.2 ± 30	0.046
Type of AFF	2 Bilateral AFFs	4 Bilateral AFFs	n.s.
Femoral neck *T*‐score	−2 ± 0.8	−1.9 ± 0.5	n.s.
Bone ALP (ng/mL)	11.9 ± 5.7	20.3 ± 9.3	0.06
P1NP (ng/mL)	28.1 ± 15	85.9 ± 34	<0.001
CTx (ng/mL)	0.186 ± 0.08	0.561 ± 0.18	0.002

Values are mean ± SD or as indicated.

AFF = atypical femoral fracture; GCC = glucocorticoid; n.s. = not significant.

### Genetic and biochemical results

**Figure 1:**
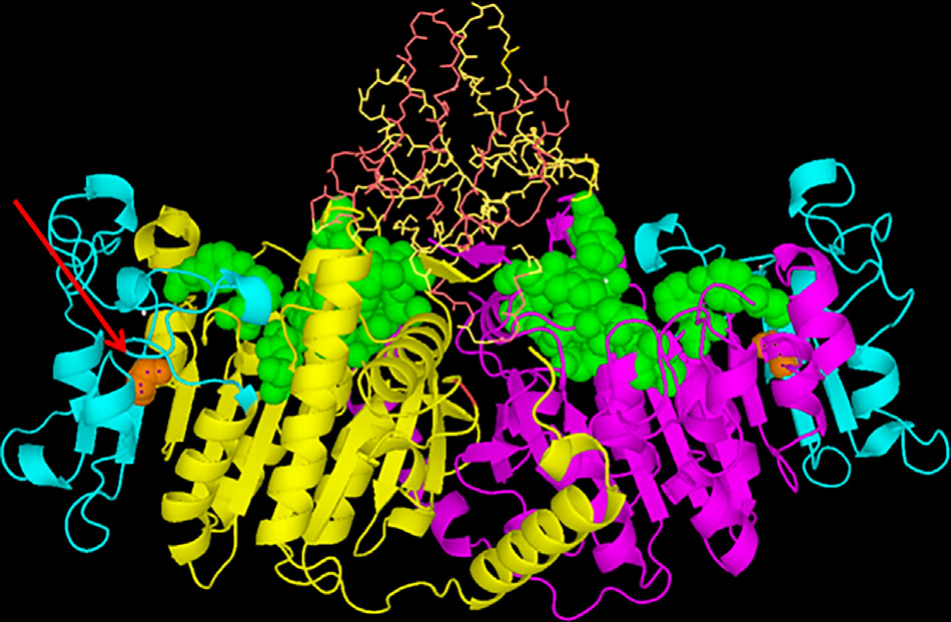
The location of the residue 288 in the 3D model of TNAP. The model is based on that described by Silvent and colleagues.^17^ Monomers are highlighted in yellow and magenta while residues of the active site are green and those of the crown domain are represented in sticks. The calcium binding site is depicted in cyan and the red arrow highlights the residue 288 in orange.

Three of the 17 patients (17.6%) presented heterozygous variants of interest: one variant in the *ALPL* gene and two in the *CYP1A1* gene. The *ALPL* variant consisted in a G to C transversion at position c.863 of the *ALPL* gene leading to the amino acid change p.Gly288Ala, which can be classified as a likely pathogenic variant. This variant has not been registered in the databases of genomic diversity Exome Aggregation Consortium (ExAC),^19^ the 1000 Genomes Project,^20^ or in disease‐associated databases (The Tissue Nonspecific Alkaline Phosphatase [TNAP] Gene Mutations Database and The Human Gene Mutation Database).^21^, ^22^ Visualization of the residue in the placental TNAP 3D model shows that it is located in the calcium binding domain (Fig. 1), which is important for the correct maintenance of structure and function of the protein.^17^

The variants identified in the *CYP1A1* gene are (i) a G to A transition at position c.407 of the *CYP1A1* gene leading to the amino acid change p.Arg136His; and (ii) a G to A transition at position c.1225 of the *CYP1A1* gene leading to the amino acid change p.Val409Ile. According to the ACMG guidelines, both variants found in the *CYP1A1* gene can be classified as a variant of uncertain significance (VUS), and the Mutation Taster bioinformatic algorithm predicted both variants to be disease‐causing (Table 2).^15^ Variant p.Arg136His has only been registered in the ExAC genomic diversity database (rs202201538) in only one allele out of 11,560 from an individual of Latino origin, wherase the p.Val409Ile variant (rs769134905) has been identified in one allele out of 3854 from an individual of the UK10K project.^23^

Only the patient with the *ALPL* likely pathogenic variant presented an increase in ALP substrates (vitamin B6 serum levels) with marginally decreased total ALP levels (Table 2) at the time of evaluation (after the AFF). In this patient treatment with teriparatide was initiated after the AFF and was maintained for 2 years; total ALP was within normal values and/or marginally decreased during teriparatide treatment. However, total ALP decreased after finishing treatment, with low values in all the subsequent determinations (4/4); serum vitamin B6 values were always increased (in 5/5 determinations [independently of teriparatide treatment]). The two patients with the *CYP1A1* VUS variants had GCC‐induced osteoporosis and had been receiving treatment with BPs for 3 and 12 years, respectively. In addition, eight of 17 patients (47%) presented polymorphisms in the *ALPL* gene; eight of 17 patients (47%) in the *GGPS1* gene, and one patient presented a polymorphism of the *CYP1A1* gene (Table 2). When we evaluated the serum levels of total ALP and/or the substrates of ALP (vitamin B6 and PEA), none of the patients with polymorphisms of the *ALPL* gene presented abnormal values (ie, decreased total ALP or increased ALP substrates).

## Discussion

The present study shows that mutations in the *ALPL* and *CYP1A1* genes may be related to the development of AFF in patients receiving BPs indicating the need to evaluate ALP substrates in patients with low total ALP levels prior to initiating BP treatment. In addition, in view of the present results the role of *CYP1A1* mutations in AFF needs further study.

In our series most patients with AFF presented with clinical characteristics similar to previous studies, such as the relatively younger age (with a mean age of 71 years) compared to that observed in the “typical” fragility hip fracture, the frequent vitamin D deficiency, the relatively higher BMD values commonly within the osteopenic range, and the frequent use of PPIs in 47% of subjects as well as concomitant GCC treatment in nearly 50% of these patients.^1^, ^2^, ^3^, ^24^ Interestingly, GCC‐treated patients showed some differential clinical characteristics compared with the remaining patients, such as younger age, more depressed bone turnover after the fracture, and a longer period of treatment with BPs previous to the fracture. Thus, although previous treatment with BPs was present in all patients, alendronate being the drug most frequently used, patients with GCC‐induced osteoporosis were treated with BPs during a mean period of 105 months, compared with 68 months in the remaining subjects. In addition, it should be noted that five of 17 subjects (∼30%) were treated with BPs for less than 5 years, which is the recommended minimal period of time proposed by several recent guidelines.^25^ Of note, one of these patients presented a mutation in the *CYP1A1* gene.

Although at present the pathophysiology of AFF is not clear, it has been suggested that prolonged BP treatment could persistently decrease cortical bone remodeling, and theoretically, alter the resolution of the stress microfractures that occurs in this location, increasing the risk for developing this type of fractures. Of interest is the similarity of fracture characteristics observed in other disorders, such as hypophosphatasia, a rare inborn metabolic bone disease caused by mutations in the *ALPL* gene.^7^ The clinical presentation of hypophosphatasia varies widely from lethal perinatal forms associated with severe mineralization defects to mild forms with scarce clinical expression in adults.^7^, ^26^, ^27^ Thus, as previously commented in the Introduction, hypophosphatasia in adults may be easily overlooked, presenting with stress fractures in the lower extremities, including diaphyseal femoral fractures.^5^, ^6^, ^8^ This entity should be suspected when total ALP serum levels are decreased, and—after ruling out other possible causes of low levels of total ALP—diagnosed by measuring the substrates of this enzyme (especially vitamin B6) and confirmed by mutational analysis of the *ALPL* gene whenever possible.^7^, ^27^, ^28^ The incidence of hypophosphatasia for severe forms in the general population is low (1/100,000 inborn). However, at present the incidence of mild versus moderate forms in the adult population is unknown, with an estimated prevalence of carriers of “moderate‐mild” mutations of nearly 1 in 6370 in the European population.^27^ Recently, it has been suggested that there may be a relatively high prevalence of asymptomatic “carriers” in 1 per 250 to 300 people.^29^ All of this indicates the need to take into account this entity, especially when evaluating antiosteoporotic treatment with BPs. Thus, BPs are analogues of calcium pyrophosphate and also inhibit the activity of APL by binding to Zn^++^ and to Mg ^++^(both needed for ALP activity).^5^, ^30^ For this reason, these drugs would be particularly contraindicated in hypophosphatasia, because they would worsen bone mineralization. In this sense, and similar to our study, Sutton and colleagues^5^ reported a patient with hypophosphatasia initially misdiagnosed with osteoporosis who developed a bilateral AFF after 4 years of treatment with BPs. Our patient was also initially misdiagnosed with postmenopausal osteoporosis and treated with oral BPs, developing the fracture after 7 years of treatment. The total ALP values were in the lower normal range at the time of evaluation, but it should be pointed out that this patient was evaluated after the fracture, when increased total ALP values can be observed and should be taken into account when evaluating these patients.^31^, ^32^ Of note, this patient showed markedly decreased total ALP levels after finishing treatment with teriparatide in all the subsequent determinations during the follow‐up, with a concomitant increase in vitamin B6 serum levels, further confirming the diagnosis of hypophosphatasia in this patient. The genetic analysis confirmed the presence of a new heterozygous and likely pathogenic variant, p.Gly288Ala, which, according to bioinformatic prediction, resulted in theoretical protein damage.^13^, ^14^, ^15^ To further study the possible pathogenicity of the variant identified we used a 3D model of TNAP with which we could locate the variant in the calcium binding domain, which is considered relevant for maintaining protein structure and function.^17^ Moreover, this variant has not been identified in the general population or in disease‐associated individuals. All of this strongly indicates that the p.Gly288Ala variant may play a pathogenic role in the manifestations of disease in this patient.

We also observed several polymorphisms in the *ALPL* gene in eight AFF patients, all of which are also described in the general population, with prevalences ranging from 1.24% to 11.1% in the European (non‐Finnish) population.^19^ None of these patients presented decreased total ALP levels nor increased ALP substrates, thereby suggesting that these *ALPL* polymorphisms do not play a pathologic role of in these cases.

Of interest were the variants detected in the *CYP1A1* gene in two patients. The MutationTaster bioinformatic algorithm predicted both variants to be disease‐causing, reinforcing the idea that these two variants may underlie disease pathogenicity. This is in concordance with the fact that these variants have not been reported in the general population (as in the case of p.Val409Ile) or have been described with a very low allele frequency (such as p.Arg136His).^19^ We also observed the presence of the pThr461Asn polymorphism in this gene in another AFF patient.

Recently, a *CYP1A1* gene mutation has also been reported in three sisters who all presented AFF after treatment with BPs.^11^ After performing whole‐exome sequencing to detect possible shared genetic variants involved in the AFF development in these sisters, the authors observed mutations in the *GGPS1* gene, thereby suggesting a possible effect on the activity of the geranylgeranyl pyrophosphate synthetase, and consequently on the effect of BPs on the mevalonate pathway. Nevertheless, they also observed a mutation in the *CYP1A1* gene, not only in the three sisters but also in an unrelated patient with AFF, further suggesting a potential role of this mutation in some cases with AFF.

Cytochromes P450 (CYP) are a major source of variability in drug pharmacokinetics and response, especially the CYP1, CYP2, and CYP3 families, which are responsible for the biotransformation of most foreign substances, including 70% to 80% of all drugs in clinical use.^10^ It should be noted that the expression of each CYP is influenced by several factors, such as genetic, regulatory cytokines, hormones, and age and sex, among others.^10^ Moreover, the *CYP1A1* gene not only encodes enzymes which catalyze many reactions involved in drug metabolism, but also the synthesis of cholesterol and steroids, the latter possibly influencing the effect of GCCs. Indeed, previous studies have shown different efficiencies in the hydroxylation of steroid hormones depending on the allelic variants on the *CYP1A1* gene, resulting in changes in its catalytic efficiency.^33^ Our two patients with the *CYP1A1* gene mutation had a GCC‐induced osteoporosis related to chronic obstructive pulmonary disease and rheumatoid arthritis, respectively, with previous fragility fractures and were also receiving treatment with BPs prior to developing the fracture. Therefore, in these particular patients we can speculate not only a possible role of these mutations in BP metabolism but also in the effect of GCC therapy. It should be noted that GCC treatment constitutes an important risk factor related to the development of AFF, with nearly 50% of the patients presenting this associated comorbidity in several series.^3^, ^24^, ^34^ Of interest, CYP1A1 has recently been shown to suppress AMP‐activated protein kinase (AMPK) signaling,^35^ which has been related to the development of osteoporosis in experimental studies. In addition, AMPK signaling has been shown to modulate the mevalonate pathway,^36^ which is also a target of bisphosphonates. All of the latter linking CYP1A1 with the bisphosphonate pathway indicates the need to perform functional studies in these *CYP1A1* mutations. Additionally, as commented on the previous page, we also observed a *CYP1A1* polymorphism in another elderly patient diagnosed with postmenopausal osteoporosis who developed bilateral AFF after 9 years of BP treatment. Of note, this particular polymorphism has been previously related to low femoral BMD and increased bone resorption, a finding attributed to an increase in the metabolism of estrogens in this population group.^37^ Nonetheless, although we cannot rule out an increased estrogen catabolism in these patients, if that were the case, we would expect a preventive effect of BPs for the bone loss related to the accelerated estrogen catabolism.

Conversely, although all patients with AFF included in this series were previously treated with BPs, none presented mutations in the *GGPS1* gene. This finding does not necessarily indicate the absence of a relationship of this gene mutation with AFF, but rather, suggests that if this were the case, it would probably be uncommon. Although we observed eight patients with a polymorphism (c.142‐6insT) in this gene, its high frequency in the European (non‐Finnish) population (33.6%),^(19)^ rules out its role in the pathogenicity of AFF. Recently, a pilot study in subjects with AFF did not observe mutations or polymorphisms in this gene.^38^

In addition, it should be pointed out that patients with AFF may also have other nongenetic factors that may predispose to this type of fracture, such as femoral and/or lower limb deformities. In this sense, recent studies have also suggested a possible contribution of proximal femoral geometry in AFF, reporting either an excessive femoral offset and femoral neck angle in varus,^39^ and/or femorotibial valgus deformities in some of these subjects.^40^

Our study, however, has some limitations, such as the relatively small number of patients, a limitation linked to AFF, a very uncommon entity; the absence of a control group and the absence of a functional study of the mutations analyzed. Nonetheless, the study also has several strengths such as the homogenous and well‐standardized population, the extensive genetic analysis, and the functional estimated evaluation of the gene mutations and polymorphisms analyzed, all of which constitute useful findings that may help to better understand the pathophysiology of this type of fracture.

In conclusion, *ALPL* and *CYP1A1* mutations may be related to the development of AFF in patients treated with BPs. The evaluation of ALP substrates in patients with low or marginally low ALP levels allowed the identification of patients with hypophosphatasia. The role of *CYP1A1* mutations in AFF clearly needs further study, not only evaluating the possible effect on BP but also on GCC metabolism.

## Disclosures

All authors state that they have no conflicts of interest.
